# Physically Crosslinked Hydrogels Based on Poly (Vinyl Alcohol) and Fish Gelatin for Wound Dressing Application: Fabrication and Characterization

**DOI:** 10.3390/polym12081729

**Published:** 2020-08-02

**Authors:** Teng Ren, Jing Gan, Liping Zhou, Hao Chen

**Affiliations:** 1 Marine College, Shandong University, Wenhua West Road, Gao Strict, Weihai 264209, China; SY20193061087@cau.edu.cn (T.R.); zhoulip@mail.sdu.edu.cn (L.Z.); 2 Beijing Advanced Innovation Center for Food Nutrition and Human Health, College of Food Science and Nutritional Engineering, China Agricultural University, Beijing 100083, China; 3 College of Life Sciences, Yantai University, Yantai 264005, China; ganjing@ytu.edu.cn

**Keywords:** poly (vinyl alcohol), fish gelatin, salicylic acid, hydrogels, wound dressing

## Abstract

We developed the interpenetrating double network composite hydrogel based on poly (vinyl alcohol) (PVA) and fish gelatin (FG) via thermal treatment and repeated freeze-thawing. A function of salicylic acid was incorporated into the hydrogel to improve its antibacterial properties. The color values, water contents, water evaporation rate, and swelling behavior were investigated. The drug-loading performance of the composite hydrogel was demonstrated by loading salicylic acid in various hydrogel systems. Moreover, the cumulative dissolution percentage of salicylic acid and the antibacterial activity of composite hydrogel were carried out. The results revealed that as FG concentration increased from 0% to 3.75% (*w*/*v*), gels changed from white to slight yellow and the swelling ratio increased from 54% to 83% (within 8 h). The presence of FG decreased the water content of gels which ranged from 86% to 89% and also decreased water evaporation rate. All gels presented the swelling index within 0.5–1.0, indicating a non-Fickian diffusion mechanism. The drug sustained dissolution behavior of pure PVA and composite hydrogel showed the same trend. Besides, the presence of the obvious bacteriostatic zones means that drug-loaded composite hydrogels have an effective antibacterial property. These results demonstrated that PVA/FG-based interpenetrating hydrogel is an appropriate biomaterial for drug-carrying wound dressing application.

## 1. Introduction

Nowadays, healthcare-associated infections have caused severe problems in clinicians, with a large number of people suffering from acute and chronic wounds. Designing new wound dressing materials has been an imperative issue in modern medical technology findings. Functional active wound dressings are expected to provide a moist wound environment, offer protection from secondary infections, remove wound exudate, accelerate tissue regeneration, and improve the efficiency of wound repair [1]. Many systems such as foams, hydrogels, and films have been developed for wound dressings in recent years. Among these wound dressing types, hydrogels are the optimal candidates because they could meet all requirements for wound healings [2].

Hydrogels are three-dimensional cross-linked hydrophilic polymers networks being used for various biomedical applications. These polymeric materials do not disintegrate in water at physiological temperature and pH but swell considerably in an aqueous medium because of their cross-linked structures [3]. Hydrogels possess many advantages such as large water content, soft elasticity, cooling effect, and so forth [4]. High water content of hydrogels assists to promote granulation and epithelialization due to the moist environment [5]. The soft elastic property of hydrogels could provide easy application and removal after wound was healed without any damage [6]. Additionally, temperature of cutaneous wounds could be lowered by hydrogels providing soothing and cooling effect [7]. Moreover, hydrogel wound dressings are non-irritant, non-reactive with biological tissue and permeable to metabolites. At the same time, hydrogels can be exploited as platforms to transport bioactive molecules e.g., antibiotics, and pharmaceuticals to wound sites [2]. The released drugs play an active role in the healing process either directly as debriding agents for removing necrotic tissue, or indirectly as antimicrobial drugs, which prevent infection and aid tissue regeneration [8].

Admittedly, hydrogels are not flawless. Single-network hydrogels have weak mechanical properties and slow response at swelling. Multicomponent networks are usually designed as interpenetrating polymer networks to address this drawback [9]. A wide variety of hydrophilic polymers have been utilized to fabricate hydrogels, including natural polymers (polysaccharides and proteins) and synthetic polymers containing hydrophilic functional groups such as –COOH, –OH, –NH_2_, and so on [10,11]. Natural polymers e.g., alginate, gelatin, chitosan [12], and synthetic polymers e.g., polyvinyl alcohol and polyvinyl pyrrolidone [13] have been widely used to fabricate hydrogels for biomedical applications.

PVA is a hydrophilic, biodegradable, and biocompatible synthetic polymer and has been widely used in different areas of the biomedical field [14]. PVA molecules would cross-linked physically under repeated freeze-thawing [15]. Compared with other synthetic polymers, PVA hydrogel possesses desirable physical properties such as good biodegradability [16], non-toxicity [17], and favorable mechanical strength [16,18], especially it is relatively cheap in contrast to many other synthetic polymers. However, single PVA hydrogel exhibits weak mechanical strength and insufficient swelling properties [19,20], which restrict its utilization as a dressing material [15]. 

Gelatin is a water soluble and biocompatible polymer, which is produced by partial hydrolysis of collagen extracted from skin and bones [21]. Among these, gelatin from marine sources such as fish skin or bone has gained attention as a base material in the biomedical scaffold, food packaging, and pharmaceutical industry because of its low cost and multi-functionality. FG possesses desirable properties such as biocompatibility, biodegradability, hydrophilia, and water retaining capacity [20,22]. In addition, FG is deemed as an ideal hemostatic and wound dressing material [23] owing to its good coagulation effect on platelets which can promote blood coagulation [24] and is also widely used for the manufacturing of hard and soft capsules, plasma expanders, and in wound care in the pharmaceutical industry. Thus, FG was elected to blend with PVA, attempting to enhance the mechanical strength and swelling response of single PVA hydrogel. 

The biggest obstacle to wound healing is infections that can lead to systemic complications [25,26]. Sustained delivery of bioactive agents was reported as highly beneficial for wounds. Salicylic acid is one of the earliest antibacterial and anti-inflammatory drugs. In the current investigation, a function of salicylic acid was to be incorporated into the hydrogels to protect its antibacterial and anti-inflammatory properties.

The objective of this work was to obtain interpenetrating polymer network hydrogels based on FG and PVA using the methods of thermal treatment and repeated freeze-thawing, attempting to modify the mechanical properties of pure FG hydrogel and the re-swelling performance of pure PVA hydrogel. The effect of FG concentration on gel properties was investigated. Then salicylic acid was added for improving the anti-microbial and anti-inflammatory properties, and the release of salicylic acid in vitro was investigated to analyze the drug-loading performance.

## 2. Materials and Methods

### 2.1. Materials

FG with model 100G NET/BAG was a kind gift from Vinh Hoan Collagen Corporation; PVA1977 was obtained from Anhui Weiwei Group Co., Ltd. (Anhui, China); analytical-grade salicylic acid was supplied by Xilong Scientific Co., Ltd. (Shantou, China); analytical-grade sodium chloride was purchased from Tianjin Ruijinte Chemical Co., Ltd. (Tianjin, China) All other chemicals are analytical grade, and without further purification unless otherwise described.

### 2.2. Preparation of Hydrogel

#### 2.2.1. Preparation of PVA/FG Composite Hydrogel

The PVA/FG composite hydrogels were fabricated by thermal treatment and cyclic freeze-thawing method [5]. In brief, PVA (10 g) was dissolved in 100 mL deionized water at 90 °C and was magnetically stirred for 1 h. Proper amounts of FG (0, 0.75, 1.50, 2.25, 3.00, 3.75 g) were then added to the solutions (80 °C) respectively under magnetic stirring, followed by ultrasonic defoaming for 20 min to remove the bubbles. Subsequently, proper amounts of these mixtures were poured into Petri dishes, followed by freezing at −20 °C for 24 h and thawing for 3 h at room temperature for four continuous cycles. Finally, the PVA/FG composite hydrogels with different FG contents were obtained.

#### 2.2.2. Preparation of Drug-Loaded PVA/FG Hydrogel

PVA/FG mixed solution with 1.50% (*w*/*v*) FG and 10% (*w*/*v*) PVA was obtained according to Section 2.2.1. Salicylic acid (0.5%, *w*/*v*) was added into the PVA/FG solution at 50 °C under magnetic stirring (for 2 h). After ultrasonic defoaming for 20 min, the composed solution was poured into the Petri dish and repeatedly frozen-thawed four times following a procedure outlined in Section 2.2.1 to obtained the drug-loaded PVA/FG composite hydrogel.

### 2.3. Characterization of Hydrogels

#### 2.3.1. Measurement of Color Values

The color values of hydrogels with different FG contents were measured by a precision colorimeter (NR110, Shenzhen Sanenchi technology co., Ltd., Shenzhen, China). The CIE Lab scale chromaticity parameters L*, a*, b* were recorded respectively. L* denotes the brightness index, L* = 0 means black, L* = 100 means white; a* denotes red and green, +a* means red and −a* green; b* denotes blue and yellow, +b* is for yellow and −b* is for blue. The color values can objectively evaluate the magnitude of the chromatic aberration and its visual difference. The implications of general color values ΔE are shown in Table 1. ΔE can be calculated by the following equation.

(1) $$\Delta E={\left[{\left(L^{*}-L\right)}^{2}+{\left(a^{*}-a\right)}^{2}+{\left(b^{*}-b\right)}^{2}\right]}^{1/2}$$

where L*, a*, and b* are the color parameter values of hydrogel sample, *L*, *a*, and *b* are the color parameter values of the control hydrogel (without FG).

#### 2.3.2. Determination of Water Content in Hydrogels

The thawing hydrogels were cut into cylinders with a diameter of 3.3 cm using a circular cutter. Then the samples were gently wiped with filter papers to remove surface water and weighted. Subsequently, they were dried in an oven (105 °C) to constant weight. The weights of all samples were recorded as the average value of three measurements. The water content of hydrogel was determined using the following equation.

(2) $$Water\;content\;\%=\frac{M_{0}-M_{1}}{M_{0}}\times 100\%$$

where *M*_0_ and *M*_1_ are the weight of the thawed hydrogel and the weight of hydrogel at dry state, respectively. 

#### 2.3.3. Swelling Properties 

The hydrogels were cut into cylindrical shaped specimens (3.3 cm in diameter) for drying in an oven to constant weight. The dried hydrogel samples were then immersed in deionized water and saline solution (0.90% *w*/*v* of NaCl in deionized water) at room temperature respectively. Subsequently these samples were taken out at the regular time intervals. The excess water on the surfaces of the samples was removed by blotting gently with filter papers and the samples were weighed immediately [27]. The swelling degree (*SD* %) was calculated as the following equation:

(3) $$SD\%=\frac{M_{t}-M_{1}}{M_{1}}\times 100\%$$

where *M_t_* is the weight at interval time *t* during water absorption, *M*_1_ is the initial weight of the dry gels.

#### 2.3.4. Swelling Kinetic Study

The water absorption character of the hydrogels in pharmaceutics resembles to the swelling of the hydrogel in polymer science. Generally, the diffusion behavior and transport of small molecules in polymers have been classified into three clear types [28], which can be distinguished by the shape of the swelling curve, represented by Equation (4).

(4) $$F=\frac{M_{t}-M_{1}}{M_{e}-M_{1}}=kt^{n}$$

*lnF* = *lnk* + *nlnt* (5)

where *M_e_* is the weight of the swollen hydrogel at equilibrium, *M_t_* is the weight of swollen hydrogel at interval times, *M*_1_ is the weight of dried hydrogel, *k* is the rate constant characteristic of the hydrogel, and *n* is the transfer exponent indicating the mechanism of swelling. According to the value of *n*, the diffusion behavior and transport of small molecules in polymers have been classified into three clear types. 

Type 1: The Fickian diffusion, where penetrate diffusion rate is the slowest and hence the diffusion controlled (*n* ≤ 0.5).

Type 2: The other extreme, where the segmental relaxation processes are the slowest and hence, the stress relaxation-controlled mechanism (*n* = 1).

Type 3: The anomalous diffusion is an intermediate situation when the penetrate mobility and segmental relaxation are on a comparable time scale (0.5 < *n* < 1).

Type 1 and type 2 can be regarded as two limiting cases of transport processes with type 3 being an intermediate case, where both processes are operative in a coupled manner. 

The slope value of *n* represents the swelling exponent. In order to clarify the diffusion process of water molecules in PVA/FG composite hydrogels, the results of the swelling measurement were used to calculate by Equations (4) and (5). The slope (*n*) of the double logarithmic plot of Equation (5) was calculated. As mentioned above, the value of *n* indicates the type of transport mechanism. When *n* ≤ 0.5, it is Fickian diffusion, when 0.5 < *n* <1, it is non-Fickian diffusion.

#### 2.3.5. Measurement of Water Evaporation Rate

The pure PVA hydrogel and PVA/FG composite hydrogel were cut into squares of 3 × 3 cm and then kept in an incubator at 37 °C (relative humidity of 50%) for 24 h. The hydrogels were taken out and weighed every 2 h until reached the constant weight. The water evaporation rate was calculated by the following equation:

(6) $$\mathrm{Water}\;\mathrm{lost}=\frac{W_{0}-W_{t}}{W_{0}-W_{d}}\times 100\%$$

where *W*_0_, *W_t_*, *W_d_* are, respectively, the initial weight, the measured weight after evaporating for predetermined time and the final weight of the hydrogels.

### 2.4. The Estimation of Drug Release In Vitro

To study the release pattern of salicylic acid from the hydrogels, a known weight of salicylic acid loaded hydrogel was immersed in 400 mL saline and placed in a thermostatic rotary shaker at shaking speed of 100 rpm at 40 °C. Subsequently the solution (7 mL) was withdrawn at 0, 3, 5, 7, 10, 15, 20, 30, 60, 90, 150 min respectively and equal volume of the same dissolution medium was added back to maintain a constant volume. The amount of salicylic acid released from the hydrogel was determined by UV-visible spectrophotometer at 290 nm and calculated from a previously calibrated standard curve. All release experiments were conducted in triplicate. 

### 2.5. Antibacterial Activity Test

The antibacterial efficacy of the salicylic acid-encapsulated hydrogel against *E. coli* (ATCC25922, Gram-positive) and *S. aureus* (CMCC (B) 26003, Gram-negative) was evaluated according to the inhibition zone. Sterile physiological saline was used to prepare various bacterial species into a suspension with a concentration of 1 × 10^6^–1 × 10^7^ CFU/mL and the concentration of bacteria was determined by a UV/VIS spectrometer (T6 new century, Beijing Puxi General Instrument Co., Ltd., Beijing, China) at 625 nm. Afterwards, 0.1 mL inoculums containing approximately 106–107 CFU/mL of tested bacteria were seeded on the surface of the solid LB media (1% tryptone, 0.5% yeast extract, 1% agar, and 1% NaCl, sterilization at 120 °C for 20 min). Finally, the salicylic acid-encapsulated hydrogels were cut into a disc form with a 6-mm diameter mold and then placed on the LB plates. The plates were incubated at 37 °C for 24 h before the diameters of inhibitory zones were measured.

### 2.6. Statistical Analysis

All the analyses were carried out at least triplicates to find mean and standard deviation values and the results were expressed as a mean ± standard deviation. Figures were generated with Origin 8.5.

## 3. Results and Discussion

### 3.1. Sensory Characteristics of Hydrogels

**Figure 1:**
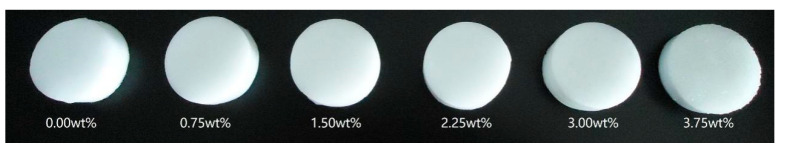
Images of the PVA/FG composite hydrogel with different fish gelatin (FG) contents.

The photographs of PVA/FG composite hydrogels with a function of FG contents are shown in Figure 1. All samples exhibited good formability. As FG concentration ranged from 0% to 3.00% (*w*/*v*), the gels showed exceptional uniformity because of the mutual compatibility of FG and PVA molecules. While as to the composite hydrogel containing 3.75% (*w*/*v*) FG, FG particles could be clearly figured out on the surface of the hydrogel. This could be attributed to the over-saturated compatibility between PVG and FG molecules at this concentration. Hence, they failed to form a uniform composite hydrogel. 

**Figure 2:**
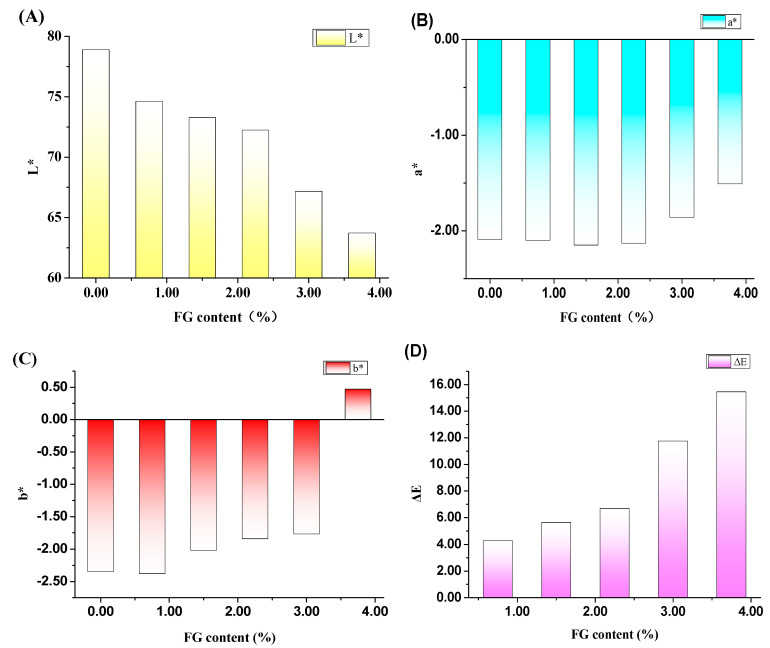
Effects of FG contents on the color of PVA/FG composite hydrogels (**A**) L*, (**B**) a*, (**C**) b*, and (**D**) ΔE, (10% PVA).

The colors of the composite hydrogels with a function of FG contents are shown in Figure 2. Owing to the color of light yellow in FG particles, the color of PVA/FG composite hydrogels gradually turned to yellow with the increasing amount of FG. Since 6.5 < ΔE < 13 denotes the distinguished color is quite obvious and 13 < ΔE < 25 means that the tested samples belong to different colors. It could be concluded that the FG contents showed great influences on the color of composite gels. When the FG content is less than 2.25%, the color differences of PVA/FG composite hydrogels are not obvious. It can be considered that these hydrogels have the same color. When the FG content is between 2.25% and 3.75%, the color difference of the gel can be clearly seen, and as the FG content exceeds 3.75%, the composite hydrogel displays a completely different color.

### 3.2. The Water Content of Hydrogels

**Figure 3:**
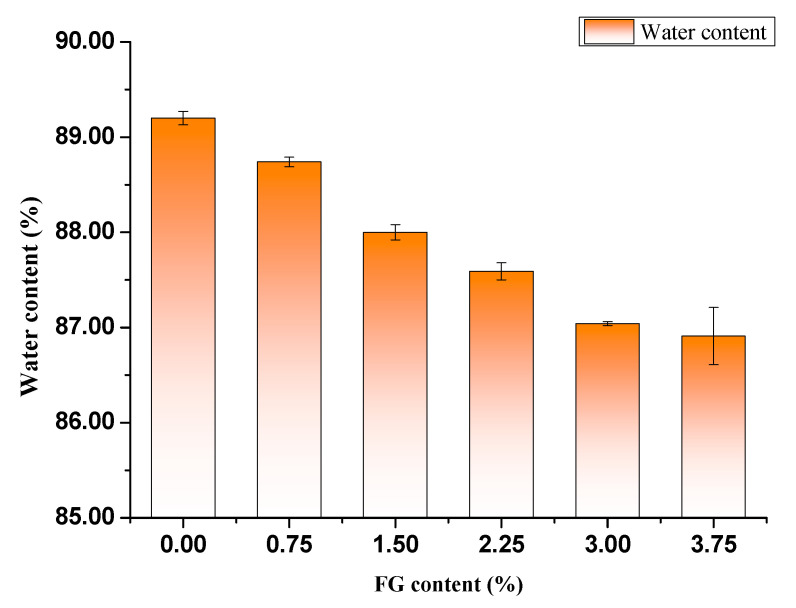
Water content of different PVA/FG composite hydrogels (10% PVA).

The water content of PVA/FG hydrogels with a function of FG amounts is presented in Figure 3. The water content of the pure PVA hydrogel was 89.2%, with the increase of FG content, the water content of the composite hydrogel decreased, but these differences were not significant, and the water content of PVA/FG composite hydrogels still ranged from 86.0% to 88.8%. There are the two possible reasons for all composite hydrogels exhibiting desirable water retention ability. The PVA molecule was abundant of hydroxyl groups which could easily form hydrogen bonds with water molecules and retain water molecules [21]. FG also exhibited strong hydrophilicity owing to a mass of hydrophilic groups of FG proteins such as carboxyl groups, amino groups, and hydroxyl groups. Consequently, compared with pure PVA hydrogel, the obtained composite hydrogels still have good water retention, they were capable of retaining enough water molecules [20].

### 3.3. Swelling Behavior Analysis

**Figure 4:**
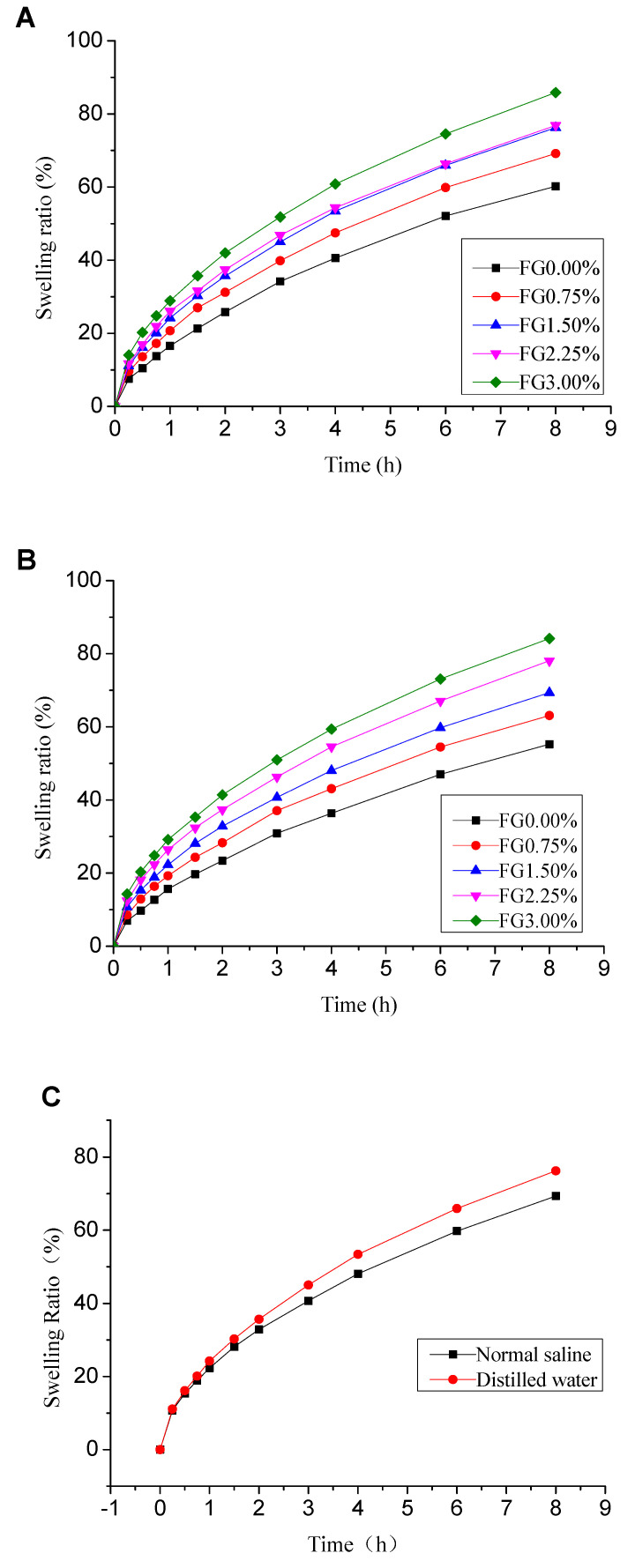
Swelling ratio of PVA/FG composite hydrogel (PVA content is 10%) in distilled water (**A**), in physiological saline (**B**) and a comparison of swelling degree of composite hydrogel (10% PVA + 1.5% FG) in distilled water and physiological saline (**C**).

One essential property of wound dressing material is good hydration capacity which facilitates rapid wound healing and proceeds to improve the re-epithelialization process [29]. Therefore, evaluation of swelling capacity of hydrogel is of great significance. This characteristic indicates the capabilities of absorbing fluids and exudates [30]. The swelling behaviors of hydrogels in distilled water as well as in normal saline were studied. Figure 4A,B displays the swelling properties of PVA/FG hydrogels with different ratios of FG in distilled water and normal saline respectively. Noteworthily, the swelling properties of hydrogels were enhanced by the presence of FG. Furthermore, the swelling property was improved steadily as FG concentration increased. The increased swelling ratio occurred because of the high solubility of FG in water [31]. Additionally, the PVA and FG molecules cross-linked each other and formed interpenetrating double network gelation under these processes, which would bring about the denser microstructure. As a result, more capillary structure could be formed to retain moisture [5,24,32,33].

A comparison of swelling degree of PVA/FG composite hydrogel (10% PVA + 1.5% FG) in physiological saline and distilled water is shown in Figure 4C. The PVA/FG composite hydrogel showed slight larger swelling ratio in distilled water than in physiological saline. This phenomenon may be caused by the different osmotic pressure of medium in which the composite hydrogel was placed [29]. The osmotic pressure of physiological saline was higher than distilled water, so the rate of diffusion of water molecules into the hydrogel in physiological saline was slower than that in distilled water. 

### 3.4. Water Evaporation Rate Analysiss

**Figure 5:**
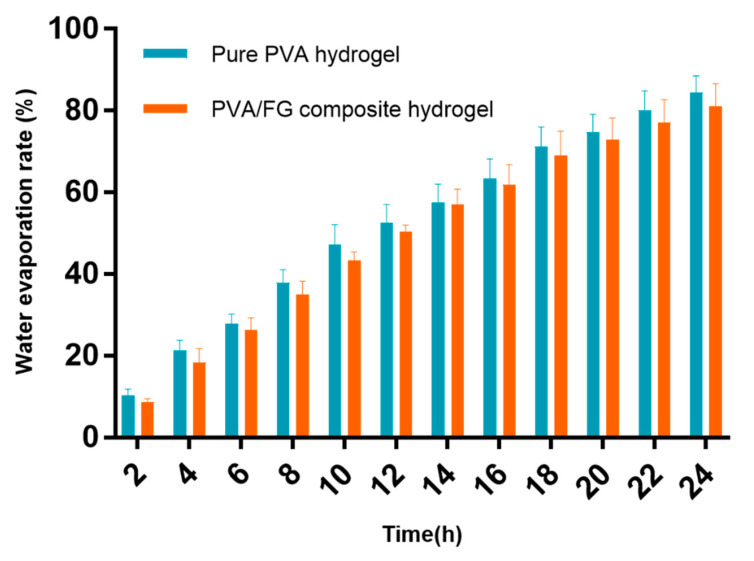
Water evaporation rate of pure PVA hydrogel and PVA/FG composite hydrogel.

Moist environment can promote wound healing, and clinical wound dressings need to be replaced repeatedly before recovering [5], as a result, the water evaporation rate of wound dressing is of importance. The hydrogel dressings with smaller water evaporation rate can reduce the replacing times leading to quicker healing, less pain, and great cost savings [34]. The water evaporation rate of pure PVA hydrogel and PVA/FG composite hydrogel is shown in Figure 5. The water loss of pure PVA hydrogel was 84.38% in 24 h while the composite hydrogels was 81.05%. This result could be ascribed to that the addition of FG increased the crosslinking density, led to denser network structure and smaller pores of hydrogel eventually [35]. The results show that the PVA/FG composite hydrogel has a better water evaporation rate than the pure FG gel, so that PVA/FG composite hydrogel is more suitable as a wound dressing compared with pure FG hydrogel.

### 3.5. Swelling Kinetics

The solvent diffusion into the hydrogel network is not passive diffusion into the void spaces of the network but includes a concomitant stretching of the network segments by advancing solvent front, which results in the plasticization of the material with a large change in the volume of the sample [24]. The swelling kinetics parameters of PVA/FG composite hydrogels with different FG contents in distilled water and physiological saline are shown in Table 2. All the values of the correlation coefficient, R^2^, were above 0.99, and with the increase of FG concentration, the values of swelling exponent *n* decreased (but not less than 0.5). This indicated the diffusion process of water molecules in PVA/FG composite hydrogels was non-Fickian diffusion [24,32]. 

### 3.6. In Vitro Salicylic Acid Release

**Figure 6:**
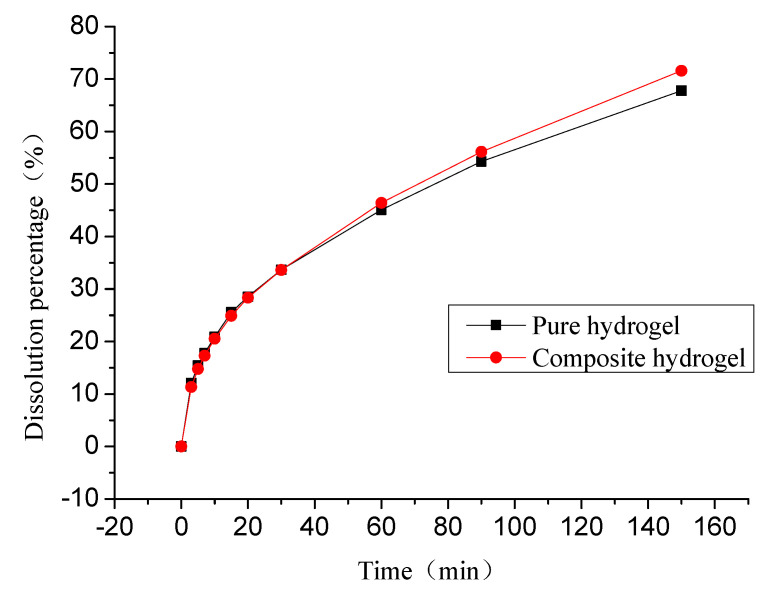
In vitro salicylic acid release profile of pure PVA hydrogel and PVA/FG composite hydrogel (1.5% FG) in normal saline.

Salicylic acid as a model drug was chosen for delivery in this study. The effect of FG concentration on the salicylic acid release behaviors from hydrogel was investigated under physiological saline. In vitro cumulative release profiles of salicylic acid from pure PVA hydrogel (10% PVA) and PVA/FG composite hydrogel (1.5% FG + 10% PVA) are exhibited in Figure 6. As seen, these two kinds of hydrogel systems showed obvious burst release in the initial 30 min, with slowly releasing in the following 2.5 h. The release percentage of pure PVA hydrogel and PVA/FG hydrogel were 33.67% and 33.63% respectively in the first 0.5 h, and 45.10% and 46.41% respectively in the first 1 h. After 3 h, the cumulative release of the composite hydrogel was slightly higher than pure PVA hydrogel, which was consistent with the change of swelling state of the composite hydrogel. This result showed that the PVA hydrogel with 1.5% FG exhibited no significant differences in the cumulative release. It could be inferred that 1.5% FG exerted little influence on the drug release behavior. The reasons for the above phenomena may be that the salicylic acid was not chemically attached to the polymer and the only interactions were intermolecular attraction and entrapment within the polymer matrix. Thus, the drug release largely depended on the swelling process and three-dimensional polymer network [32]. 

### 3.7. Antibacterial Activity

**Figure 7:**
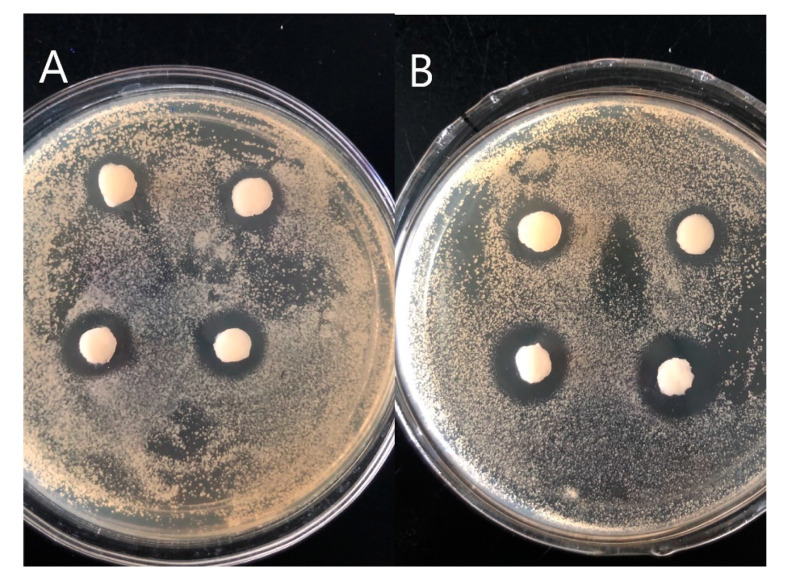
Inhibitory effect against *E. coli* (**A**), *S. aureus* (**B**).

Antibacterial activity is an important property of hydrogel dressings. We evaluated the antibacterial activity of PVA/FG composite hydrogels loaded with 0.5% salicylic acid by observing the range of the zone of inhibition. From the photos shown in Figure 7, the presence of the obvious bacteriostatic zone represents that composite hydrogels encapsulated with salicylic acid exhibited good antimicrobial activity against Gram-positive bacteria (*S. aureus* CMCC (B) 26003) and Gram-negative bacteria (*E. coli* ATCC 25922). The largest average diameters of inhibition zone were even 17 mm. Consequently, the PVA/FG composite hydrogel encapsulated with salicylic acid has good antibacterial effect and can be used as a wound dressing.

## 4. Conclusions

In this research, PVA/FG interpenetrating polymer network hydrogels were synthesized by thermal treatment and cyclic freeze-thawing method and salicylic acid release test was used to demonstrate the excellent in vitro drug release behavior of PVA/FG composite hydrogels. The FG content showed significant influences on the apparent characteristics, color values, swelling ratio, water evaporation rate, and swelling kinetics of PVA/FG composite hydrogel and discovered that the dose of FG was the key factor in obtaining PVA/FG hydrogels with desirable properties. Additionally, the composite hydrogels exhibited high capability in absorbing fluid based on the swelling behavior. The study of the swelling kinetics revealed that the swelling process of the composite hydrogel was not influenced by the addition of FG. The results of in vitro model drug salicylic acid release showed that the PVA/FG composite hydrogels had good sustained release property. Moreover, the antibacterial activity test of PVA/FG hydrogels showed their good antibacterial effect. As a result, the PVA/FG hydrogels showed excellent physical properties, which met the essential requirements for ideal medical applications. Thus, it is a potential wound dressing with excellent forming and physical properties.

## Figures and Tables

**Table 1:** General color values and their corresponding implications.

Color Values	Implications
1.6 < ΔE < 3.2	Cannot distinguish its color difference
3.2 < ΔE < 6.5	A few people can tell the difference in colors
6.5 < ΔE < 13	The color difference is very obvious
13 < ΔE < 25	Most belong to different colors
ΔE > 25	Different colors

**Table 2:** *n* values (swelling exponent) for composite hydrogels at various FG contents in distilled water and normal saline (PVA content is 10%).

FG Content (%)	Distilled Water		Normal Saline	
	*n*	R^2^	*n*	R^2^
0.00	0.6028	0.9977	0.6130	0.9986
0.75	0.5832	0.9991	0.5795	0.9997
1.50	0.5631	0.9996	0.5444	0.9998
2.25	0.5454	0.9990	0.5276	0.9999
3.00	0.5255	0.9999	0.5141	0.9999
